# A163 INFECTION WITH HYMENOLEPIS DIMINUTA ELICITS A TH2 IMMUNITY AND ALLEVIATES DNBS-COLITIS IN A MOUSE MODEL WITH MINIMAL INTESTINAL MICROBIOTA

**DOI:** 10.1093/jcag/gwac036.163

**Published:** 2023-03-07

**Authors:** S Li, A Wang, D M McKay

**Affiliations:** Physiology and Pharmacology, University of Calgary, Calgary, Canada

## Abstract

**Background:**

Immunocompetent mice infected with the rat tapeworm *Hymenolepis diminuta* are protected from dinitrobenzene sulphonic-acid (DNBS)-induced colitis that was accompanied by the increased relative abundance of short-chain fatty acid (SCFA)-producing bacteria and fecal SCFA levels at 8 days post-infection (dpi). These changes were not observed in antibiotic-treated BALB/c mice or germ-free mice. To further characterize the host-parasite-microbiota interaction in the control of colitis, experiments have been conducted in a reductionist system in which the *H. diminuta* anti-colitic response was tested in mice colonized with 12 commensal bacteria representing the major five phyla of the mouse gut.

**Purpose:**

To identify key bacterial species and microbe-derived mediators involved in the *H. diminuta*-induced protection from DNBS-induced colitis.

**Method:**

OligoMM^12^ (Oligo-Mouse-Microbiota) mice (n=4-8/group) were infected with 10 viable antibiotic-treated *H. diminuta* cysticercoids eight days prior to challenge with DNBS (3mg, i.r.). Mice were monitored daily and necropsied 72h post-DNBS. Disease was assessed by a macroscopic disease activity score, colon length and histopathology on H&E sections of mid-colon. Production of IL-4, -5, -10 and -13 by concanavalin-A treated spleen cells by ELISA was used as an index of successful helminth infection.

**Result(s):**

OligoMM^12^ mice treated with DNBS developed acute colitis, with 3/8 mice requiring humane euthanization, while all mice infected with *H. diminuta* survived the DNBS challenge. Successful infection was confirmed by statistically significant increases in splenic output of the Th2-cytokines in response to mitogenic stimulation and efficient expulsion of the worms burden by 11 dpi. The severity of DNBS-induced colitis was reduced in *H. diminuta*-infected mice (n=4), although additional experiments are required to confirm the statistical significance of these data.

**Conclusion(s):**

Reproducible DNBS-induced colitis in mice with the simplified OligoMM^12^ consortium of bacteria and reduced disease in *H. diminuta*-infected mice has created a model by which the participation of exact bacterial groups or species in *H. diminuta*-induced suppression of colitis can be tested. This is of translational value, since the failure of helminth therapy in IBD could be predicated by dysbiosis in the patient, and therefore potentially overcome by combination therapy of ‘probiotic’ and helminth.

**Please acknowledge all funding agencies by checking the applicable boxes below:**

CIHR, Other

**Please indicate your source of funding;:**

NSERC-CGS D, Eyes High doctoral Recruitment Scholarship

**Disclosure of Interest:**

None Declared

